# A Survey of African American Physicians on the Health Effects of Climate Change

**DOI:** 10.3390/ijerph111212473

**Published:** 2014-11-28

**Authors:** Mona Sarfaty, Mark Mitchell, Brittany Bloodhart, Edward W Maibach

**Affiliations:** 1 Center for Climate and Health, George Mason University, 4400 University Drive, MS 6A8, Fairfax, VA 22030, USA; E-Mails: bbloodha@gmu.edu (B.B.); emaibach@gmu.edu (E.W.M.); 2 Commission on Environmental Health, National Medical Association, Silver Spring, MD 20910, USA; E-Mail: mmitchell@enviro-md.com

**Keywords:** climate change, health impacts, medical practice, environmental health, climate and health, vulnerable populations, African Americans

## Abstract

The U.S. National Climate Assessment concluded that climate change is harming the health of many Americans and identified people in some communities of color as particularly vulnerable to these effects. In Spring 2014, we surveyed members of the National Medical Association, a society of African American physicians who care for a disproportionate number of African American patients, to determine whether they were seeing the health effects of climate change in their practices; the response rate was 30% (*n* = 284). Over 86% of respondents indicated that climate change was *relevant to direct patient care*, and 61% that *their own patients were already being harmed by climate change moderately* or *a great deal*. The most commonly reported health effects were *injuries from severe storms, floods, and wildfires (88%), increases in severity of chronic disease due to air pollution (88%), and allergic symptoms from prolonged exposure to plants or mold (80%)*. The majority of survey respondents support medical training, patient and public education regarding the impact of climate change on health, and advocacy by their professional society; nearly all respondents indicated that the US should invest in significant efforts to *protect people from the health effects of climate change* (88%), and to *reduce the potential impacts of climate change* (93%). These findings suggest that African American physicians are currently seeing the health impacts of climate change among their patients, and that they support a range of responses by the medical profession, and public policy makers, to prevent further harm.

## 1. Introduction

The Third National Climate Assessment: *Climate Change Impacts in the United States* (NCA3) [1], published in May 2014, reported that health effects of global climate change are already evident in the United States. The Fifth Assessment Report of the International Panel on Climate Change (IPCC AR 5), released a few months earlier in 2014, reached similar conclusions about the health impacts of climate change around the world [2]. The risk of adverse respiratory effects due to poor air quality, health problems caused by heat events, and injuries attributable to extreme weather are found in the U.S. already and are likely to increase in the future. The spread of food-borne, vector-borne, and waterborne diseases, and harms to mental health are additional likely by-products of climate change.

The NCA3 and IPCC AR5 reports—which were both based on a substantial number of peer-reviewed studies—offer clarity and consistency about health problems caused or worsened by climate change and raise an obvious and pressing question: Are the health problems associated with climate change being witnessed by practitioners of clinical medicine? If these problems are identifiable to researchers, it is reasonable to assume that practitioners would be seeing them. To answer this question, we surveyed members of a U.S. medical society to determine if a group of physicians in a likely position to witness the health effects from climate change were actually witnessing them in their practices, and to assess what actions, if any, they feel the medical community should be taking to address the problems.

The research was a collaborative effort between the National Medical Association (NMA) and the George Mason University Center for Climate Change Communication (Mason 4C). NMA is the U.S. nonprofit medical association that has as its core mission representing African American physicians and their patients. It is dedicated to improving public health, eliminating health disparities and promoting parity in the practice of medicine. NMA members care for patients of all races and ethnicities, but as a group have a larger percentage of patients who are people of color than other medical societies. These patients are vulnerable for such reasons as low income or assets, environmental or social injustice, and pre-existing chronic disease. There is a growing literature documenting the disproportionate impact of climate change on the health of vulnerable people and communities [1,3,4,5]. Thus, physician members of NMA may have a key vantage point from which to observe possible health effects and consider needed actions.

The current survey was collaboratively designed by researchers from the Mason 4C Program on Climate and Health and the NMA Commission on Environmental Health. Mason 4C conducts an extensive program of survey research with members of the public and various professional audiences [6,7,8]. 

## 2. Methods

### 2.1. Survey Instrument

The survey instrument was developed to assess physicians’ experiences with climate change, particularly whether they were witnessing health effects in their own patients. The instrument also included nonclinical questions on climate change beliefs and policy preferences that were drawn from earlier surveys with members of the general public [7,8]. The questions regarding clinical observations were developed by the research team and piloted with clinicians from the two partnering organizations as well as other medical societies that are interested in conducting similar assessments amongst their own members. The 15 to 20 min survey included a total of 65 questions, including open-ended questions that invited respondents to provide clinical anecdotes about their patients, barriers they face to discussions about climate and health with patients, and additional resources they would find useful. As described below, the survey was initially administered via paper and pencil, and subsequently online. A complete list of survey questions can be downloaded from the Mason 4C website [9]. The survey was approved by Mason’s Institutional Review Board (Project 563778-1/2).

### 2.2. Contact Procedures

The survey was conducted in March and May of 2014. Paper surveys were completed by the attendees of the 16th NMA National Colloquium on African American Health, which is a policy conference attended by national, state, and local NMA leaders, as well as a few leaders of other health and civic organizations, held in March of 2014. Subsequently, in May 2014, other members of NMA were emailed an online version of the survey.

The paper surveys were distributed during a session of the Colloquium. The survey was introduced by a member of the NMA, outlining the importance of gaining physicians’ perspectives on climate change and health. Respondents were entered into a raffle to win an iPad mini. These respondents were presented with the preliminary findings of the survey at a subsequent session of the Colloquium, two days later.

The second round of surveys was distributed via email in an online format. Initially, an emailed letter of request was sent to all NMA members with emails on record who had attended the previous year’s NMA Annual Convention and had agreed to receive emails from the NMA; a link to the online survey was embedded in that request. Three reminders, over the course of two weeks, were sent to members of the sample who had not yet responded. To encourage participation, several participation incentives were offered on a lottery basis: a 2015 NMA membership; a free hotel stay for the upcoming Annual Convention; and two department store gift certificates. For individuals who previously had taken the survey at the National Colloquium, there was an option on the consent page that took them immediately to the end of the survey, so they would not take the survey a second time.

Before taking the survey, participants were told the purpose of the study was to assess their attitudes about how climate change is related to health. For the initial survey administration, this information was announced by an NMA member and included in the informed consent. For the online survey, this information was included in the letter of request and part of the informed consent.

### 2.3. Analysis

Descriptive statistics were run on all variables, using unweighted data. The data were analyzed using SPSS statistical software. All mean differences reported are deemed significant if *p* < 0.05 in a two-way test. Confidence intervals (CI’s) were calculated using an online CI calculator [10]. Based on the sample size, the approximate confidence intervals for any survey question was ± 5.8%, therefore any response items that differ less than 8.5% (the square root of the sum of the squares of the differences) should not be considered statistically different. Data was not weighted, but responses were segmented by region and were not statistically different from each other when calculated using ANOVA. No regions of the U.S. (Northeast, South, Mid-West, Southwest, and Pacific Northwest) or coastal location (states on a coastline *vs.* landlocked states) differed significantly from the overall national responses. Open-ended comments were edited for minor grammar and spelling corrections, and some comments that did not directly address the question were removed from this report; personally identifying information included in responses is also not reported.

## 3. Results

### 3.1. Sample

Nearly all respondents (95%) were African American. More women (60%) than men (40%) completed the survey, and the large majority of participants (80%) were between 31 and 65 years old, with those under 30 making up only 5% of the sample, and those over 65 making up 15%. Most respondents hold an M.D. degree (81%) or an M.D. plus another degree (13%).

Respondents’ work was overwhelmingly clinical, with 78% practicing mainly ambulatory or hospital-based medicine. Over half were practicing primary care (internal medicine, pediatrics, family practice, or obstetrics and gynecology) and more than 40% were in other specialties. The majority of respondents worked in urban or suburban areas, with only 5% working in rural areas and another 4% working in a mix of these areas. Respondents practiced in 33 U.S. states, varying in population size, geography, and political leaning. States (or jurisdictions) with at least five respondents include California (20), the District of Columbia (14), Florida (10), Georgia (26), Illinois (7), Indiana (5), Louisiana (9), Maryland (24), Massachusetts (9), Michigan (8), Missouri (8), New Jersey (5), New York (18), North Carolina (9), Ohio (9), Pennsylvania (6), Tennessee (7), Texas (17), Virginia (9), and Wisconsin (6). Respondents indicated a diverse mix of patients by race (White and Non-White) and ethnicity (Latino and Non-Latino) although their patients were disproportionally non-white, and had a mix of insurance types.

### 3.2. Response Rate

At the Colloquium in March 2014, 101 out of 139 surveys distributed were completed. The online survey went to 999 NMA members; 67 bounced back because of incorrect e-mail addresses. Of the 932 physicians who did receive the emails, 200 accessed the survey online. Seventeen online responses were dropped because respondents chose not to participate or indicated that they had already taken the survey in person at the Colloquium. The total number of responses for the two surveys was 284, for a total response rate of 30%. Since every survey participant did not answer every question; total response numbers are presented for each question in the supplementary materials.

### 3.3. Beliefs, Knowledge, and Extra-Clinical Experience

**Figure 1:**
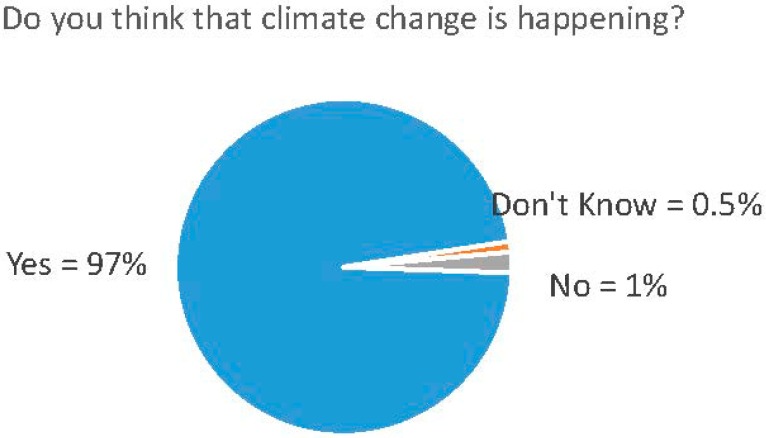
Proportion of African-American physicians who understand climate change is happening.

A solid majority of respondents are convinced that human-caused climate change is occurring. Over 97% of respondents indicated that climate change is happening (see Figure 1), and 62% said that it is mostly or entirely caused by human activity.

Nearly all survey respondents reported that climate change is relevant to direct patient care (88%) (A great deal 24%, A moderate amount 42%, Only a little 22%), suggesting they think that climate change can contribute to observable health effects that require medical treatment. Only a minority reported being “very” (6%) or “moderately” (18%) knowledgeable about the association between climate change and health impacts; conversely, many reported being only “modestly” (48%) or “not at all” knowledgeable (28%). Nearly half of respondents indicated that, outside of their role as a health professional, they had personally experienced climate change “a great deal” (10%), or a “moderate amount” (38%); others indicated “only a little” (40%), “not at all” (7%), or “don’t know” (7%.).

### 3.4. Clinical Experience

Respondents reported that climate change is affecting the health of Americans and their own patients. A majority of respondents indicated that, over the past decade, climate change has “harmed” people in their city or county “a great deal” (20%) or a “moderate amount” (46%), while relatively fewer indicated “only a little” (20%) or no harm (2%); 12% indicated “don’t know.” Most respondents also reported that climate change is affecting the health of *their own* patients “a great deal” (18%) or a “moderate amount” (43%), while fewer responded “only a little” (18%), “not at all” (3%) or “don’t know” (10%). A small number (8%) indicated that they don’t currently see patients.

The most common health effects of climate change that respondents observed among their patients were injuries due to severe weather (88%), air pollution-related increases in severity of chronic disease (88%), increased allergic symptoms (80%), and heat related effects (75%). Other conditions affecting their patients included vector-borne infections (Lyme disease or West Nile Virus) (58%), diarrhea from food/waterborne agents of infection (56%), and mental health problems related to these health issues (40%). Respondents expected to see increased clinical events in these categories in the next 10 to 20 years compared to what they are currently seeing. Increases in estimated changes over time were significant for heat effects, vector-borne infections, and diarrhea from food/waterborne illnesses; see Table 1.

**Table 1:** Responses to the question: In which of the following ways, if any, do you think your patients are currently being affected by climate change, or might be affected in the next 10–20 years?

Time Period	Yes	No	Don’t Know	N
Injuries due to severe storms, floods, droughts, fires				
Now	88%	5%	8%	240
10–20 years from now	90%	1%	9%	219
Air pollution related increases in severity of illness				
Now	88%	3%	10%	242
10–20 years from now	91%	2%	8%	221
Increased care for allergic sensitization & symptoms of plant/mold exposure				
Now	80%	8%	13%	240
10–20 years from now	86%	4%	11%	222
Heat-related effects				
Now	75%	11%	14%	236
10–20 years from now	88%	3%	9%	221
Vectorborne infection				
Now	58%	16%	26%	232
10–20 years from now	70%	9%	21%	222
Diarrhea from food/waterborne illnesses				
Now	56%	22%	22%	238
10–20 years from now	67%	10%	23%	222

Respondents were invited to provide an anecdote describing their own experience of climate change health impacts with a patient; these are summarized in Section 3.9 below.

### 3.5. Affected Groups

A large majority of respondents indicated that *certain groups* of people will be disproportionately affected by climate change, including people with chronic diseases (88%), people living near or below the poverty line (86%), young children ages 0–4 (83%), adults over age 60 (80%), and people of color (73%).

### 3.6. Current Preparedness, Barriers to Engagement, and Perceived Value of Resources

While half of respondents indicated their primary hospital is well prepared to respond to climate-related events—with 35% agreeing and 15% strongly agreeing with the statement *“The primary hospital that I admit to is well prepared for climate-related events (e.g., disasters/emergencies, extreme weather events, increases in certain diseases, etc.)”*—the other half had no opinion or disagreed (30% were neutral and 20% disagreed).

Fewer respondents indicated that “My primary place of work is doing an effective job minimizing its use of fossil-fuels (e.g., conserving energy/water, recycling equipment, *etc.*)”; only 6% strongly agreed and 23% agreed with this statement, while 7% strongly disagreed, 34% disagreed, and 30% were neutral.

Significant barriers to engaging with patients on the issue were identified. Respondents’ lack of knowledge about how to approach the issue with their patients (71%), and their lack of time (69%) were found to be the most common barriers. Less common barriers included non-billability of time spent speaking with patients about it (39%), patient disinterest (27%), and lack of perceived impact (22%).

A large majority of respondents agreed that each of three specific resources would be helpful to them: *“continuing medical education (CME) on climate change and health”* (41% strongly agree, 48% agree); *“patient education materials”* (41% strongly agree, 45% agree); and *“policy statements provided by my professional associations”* (34% strongly agree, 48% agree).

Many respondents provided open-ended suggestions of other resources that would be helpful to them; the majority of these suggestions focused on advocacy and alternate forms of education. For advocacy, responders suggested a greater presence of local professional associations involved in the issues and working to influence policy, community advocacy, public service announcements, and use of social media. For useful alternate forms of education, responders suggested webinars, videos, and podcasts, as well as presentations in the community at public forums and churches, and patient education materials made available at churches to inform the public about the health effects of climate change.

### 3.7. Preferences for Physician Response to Climate Change and Self-Efficacy

The majority of survey respondents indicated that physicians and medical societies have a role to play in addressing climate change and health, including education of doctors, patients and the public, as well as leadership and advocacy; see Table 2. A large majority also expressed a moderate (53%) or strong sense (25%) of self-efficacy to contribute to effective action on climate change.

**Table 2:** African American physicians’ response preferences and self-efficacy for dealing with climate change as a health issue.

	Strongly Agree	Agree	Neutral	Disagree	Strongly Disagree
Physicians should have a leadership role in encouraging offices, clinics, hospitals to be as environmentally sustainable as possible.	34%	47%	16%	3%	0.5%
My medical societies should have a significant advocacy role in relation to climate change and health.	28%	48%	19%	5%	1%
Physicians have a responsibility to bring the health effects of climate change to the attention of the public.	25%	46%	23%	5%	0.5%
Physicians have a responsibility to bring the health effects of climate change to the attention of their patients.	24%	51%	20%	5%	0%
I feel that actions I take in my personal and/or professional life can contribute to effective action on climate change.	25%	53%	17%	6%	0%
Teaching about climate change and its association with health impacts should be integrated into medical education.	30%	50%	12%	5%	3%

### 3.8. Support for Broader Public Policy

Nearly all respondents feel the U.S. should take significant steps to reduce climate change (through prevention) and protect people from its harmful effects (through preparedness). Specifically, in response to the question *“How big of an effort should the U.S. make to reduce the potential impacts of climate change (i.e., prevention)?”* a majority (55%) indicated “a large-scale effort, even if it has large economic costs”, while 38% indicated “a medium-scale effort, even if it has moderate economic costs,” and 7% indicated “ a small-scale effort, even if it has small economic costs;” only 1% indicated “no effort.” This question was intended to assess respondent’s preferences regarding the magnitude of climate change mitigation responses in terms that would be recognized by medical practitioners. Similarly, in response to the question *“How big of an effort should the U.S. make to protect people from the potential harmful health effects caused by climate change (i.e., preparedness)?”* a majority (56%) indicated “a large-scale effort, even if it has large economic costs”, while 37% indicated “a medium-scale effort, even if it has moderate economic costs”, and 7% indicated “ a small-scale effort, even if it has small economic costs”; only 1% indicated “no effort.” This question was intended to assess respondent’s preferences regarding the magnitude of adaptation responses, but in familiar terms used to discuss medical care.

### 3.9. Respondent Anecdotes

Respondents offered many brief anecdotes about their experiences with patients; see Table 3. 

**Table 3:** Selected answers in response to the question, “Please describe if you have a relevant anecdote about a patient who has experienced one of these impacts”.

With the aging of the population, the incidence of heat strokes has risen in my practice area.
Development of allergy after hurricane
Extreme weather (heat and dry climate) causing heat strokes and brush fires, with subsequent smog (and) worsening of asthma symptoms
I had a patient who had a severe respiratory infection. His family had the same infection. They were housebound due to Hurricane Sandy. This delayed their medical care.
I have many patients with asthma induced by weather changes and this has been increasing over the last few years
In New Orleans there are a lot of patient’s who experience severe symptoms from asthma. This was a prevalent concern since we are surrounded by two large bodies of water. However following Katrina and it’s damage now mold has become an unwelcome presence in a lot of patient’s lives.
Increased heat and dry air cause increased blowing dust, and more Upper Respiratory allergy, and irritant Symptoms
My patient experienced atrocities during hurricane Katrina. As a result, she had PTSD and severe depression that prevented her from holding a stable job. I do believe that with climate change and global warming, we should expect more hurricanes of Katrina’s severity and such resultant mental health issues.
My practice works with injury and the area in which I work has been affected by flooding and increased snow fall both as a consequence of climate change, which has increased episodes of back injury from snow removal and water removal
The severe weather and snow have limited patient access to the doctor. This causes acute problems to become chronic problems and therefore much more difficult to treat.
Weather related increases in COPD exacerbation, cardiac failure exacerbations, Sickle crises, asthma….

## 4. Discussion

This survey demonstrates that African American physicians in the National Medical Association understand that climate change is happening; 97% hold this view. This stands in contrast to public understanding that climate is happening–which in Spring 2014 was 64% [7] but is similar to the 96% rate found among physician members of the American Academy for the Advancement of Science (most of whom were academic research scientists) in 2008 [11].

Several possible interpretations of this finding should be considered. As physicians, trained in the biomedical sciences working in disciplines shaped by scientific evidence, participants might have a greater appreciation for the significance of a global consensus on the part of climate scientists and national academies of science about the reality of climate change. The evidence for this is limited given that only 29% of respondents thought the consensus of climate scientists was over 80%, when, in fact, it is 97% or greater [12]. Since the initial group of participants heard the preliminary results of the survey at the March Colloquium, this could have influenced the second group of participants. While it is impossible to know for sure that this did not happen, it is unlikely that these busy physicians shared influential information with other members. In addition, this seems unlikely to alter their medical judgment about the health problems of their own patients.

Another possible explanation is that people who experience the effects of climate change are more likely to be convinced that it is happening [13,14]. The large majority of survey participants (88%) responded that outside their clinical work, they had personally experienced some degree of climate change; this stands in stark contrast to the rate at which members of the American general public feel that they have personally experienced climate change—35% in 2014 [7]. African American physicians may be more attuned to having personally experienced climate change because they may practice in locations where change has been more apparent, or because they take care of people who are more vulnerable to climate change’s health effects. Sixty-nine percent of respondents had a primary work setting within an urban area where urban heat island effects, poor air quality, and the synergy between the two may be factors that affected patient experience and thus practitioner opinion. In addition, NMA members care for disproportionate numbers of Medicaid and Medicare recipients and other vulnerable people with lower incomes, and higher rates of chronic diseases.

Although the participation rate in the survey was high for a physician survey (30%), one needs to show caution in generalizing the results. We surveyed only NMA members; the majority of African American physicians in the U.S. are not NMA members.

Conversely, there are reasons to suggest that our findings are at least generally indicative of the experiences and views of African American physicians in the U.S. Our respondents work in 33 different states of various sizes, indicated a range of political leanings, and included a strong mix of clinical specialists (over 40%) as well as primary care practitioners (53%). The health effects identified overlap appreciably with the responses to the open-ended question; and both reflect the health issues described in the U.S. National Climate Assessment, specifically injuries from extreme weather, air pollution-related increases in the severity of illness for lung and cardiovascular disease; heat-related effects; and increased allergic sensitization and symptoms [1]. Lastly, the practice locations of respondents are consistent with their observations: 69% of respondents practice in urban areas where heat plus air pollution present a double threat to many vulnerable people (heat is a threat in the most rural areas also). Longer ragweed pollen seasons found in much of the country (urban, suburban, and rural) further exacerbate the problem. 

Respondents expressed strong support for policy solutions at the national level to reduce the impact of climate change and protect and prepare the population. Moreover, they feel strongly that physicians and their associations should assume a leading role in advocating for sustainability as well as measures that address the health effects of climate change. In support of these approaches, 82% would like policy statements from their professional associations. Since many physicians belong to more than one professional association, this opinion may be a key to empowering physician communities to become involved. The respondents demonstrated a strong sense of self-efficacy in that they believe that their own actions—in their professional lives and personal lives—can make a difference. Research supports the key role of self-efficacy as a necessary condition for climate change action [15].

Respondent’s assessment of their own knowledge base also provides direction for future work. Only one quarter of survey respondents feel very or moderately knowledgeable about the association between climate and health impacts, while almost half feel only modestly knowledgeable. If physicians are going to follow through on the actions favored by large majorities, including having physicians educate their patients and the public about the connections between climate change and health, then continuing education about the relationship between climate and health is a pressing need. Overwhelmingly, respondents identified continuing medical education, undergraduate medical education, and patient education materials as needs.

## 5. Conclusions

There is a near consensus among African American physicians in the National Medical Association that climate change is occurring, and that its impacts are relevant to direct patient care. A majority of these medical practitioners have concluded that they are already encountering health impacts of climate change in their practice—most notably injuries due to extreme weather, health effects of hotter temperatures, detrimental impacts on chronic diseases due to air pollution, and more allergy problems—and they anticipate that some of these problems will increase in the next 10 to 20 years. They believe that physicians should inform their own patients and the general public about the health risks created by climate change, and that medical societies should have a significant advocacy role. They strongly support education at all levels of medical education, including undergraduate and continuing medical education (CME), and favor public education and patient education materials. Moreover, these African American physicians support government efforts to protect and prepare people and to address the problem.

These findings suggest avenues for continued work and study. Clinicians who believe it is their responsibility to educate their patients and the public should be enabled to do so through the development of appropriate educational resources. Other relevant medical/clinical societies should assess the experiences and preferences of their members to determine the extent to which they are similar to, or different than, those found among NMA members. If the current pattern of findings are found more broadly through the medical community, the medical education community should determine if changes are needed in undergraduate education and CME, and medical associations should consider what roles they wish to play in educating the public and policy makers, and in advocating for preventive and protective measures.

Few members of the public and policy makers have personal contact with a climate scientist, but most people have contact with—and trust—physicians and other health professionals. Given this, and the profound health consequences associated with climate change, the medical and health communities may have a special role to play in helping individuals, communities, and nations come to terms with climate change, and make informed decisions about how to respond. Of course, funding would be needed to support such activities; funders should consider how they can support the climate and health efforts of the clinical and public health community.

## Supplementary Files

Supplementary File 1
