# Supplementary material for: A Survey of African American Physicians on the Health Effects of Climate Change
Source: Int J Environ Res Public Health. 2014 Nov 28;11(12):12473–85. doi: 10.3390/ijerph111212473 (PMC4276625; doi:10.3390/ijerph111212473)
Supplement: Supplementary File 1 [file ijerph-11-12473-s001.pdf]

Supplementary Information

A Survey of African American Physicians on the Health Effects of Climate Change

Section A

Climate change refers to the idea that the world's average temperature has been increasing over the past 150 years, may be increasing more in the future, and that the world's climate is changing as a result.

What do you think: Do you think that climate change is happening?

(Those that answered YES were then asked) How sure are you that climate change is happening?

(Those that answered NO were then asked) How sure are you that climate change is not happening?

Response Options	Percent Response	Response Number (N)
**Yes**	**97%**	**271**
Extremely Sure	31%	93
Very Sure	35%	107
Somewhat Sure	20%	59
Not at all Sure	2%	5
**Don't Know**	**2%**	**5**
**No**	**1%**	**3**
Not at all Sure	0.7%	2
Somewhat Sure	0%	0
Very Sure	0%	0
Extremely Sure	0.3%	1
**Total**	**100%**	**279**

Do you think climate change over the past 150 years is…

Response Options	Percent Response	Response Number (N)
Caused entirely by human activities	4%	10
Caused mostly by human activities	58%	161
Caused about equally by human activities and natural changes in the environment	34%	96
Caused mostly by natural changes in the environment	3%	8
Caused entirely by natural changes in the environment	1%	2
None of the above because climate change isn't happening	0.5%	1
**Total**	**100%**	**278**

To the best of your knowledge, what percentage of climate scientists has concluded that human-caused climate change is occurring?

Response Options	Percent Response	Response Number (N)
0–20%	2%	6
21%–40%	12%	33
41%–60%	17%	46

61%–80%	24%	67
81%–100%	29%	81
Don't Know	17%	46
**Total**	**100%**	**279**

How knowledgeable do you feel about the association between climate change and health impacts?

Response Options	Percent Response	Response Number (N)
Very knowledgeable	6%	16
Moderately knowledgeable	18%	51
Modestly knowledgeable	48%	132
Not at all knowledgeable	28%	79
**Total**	**100%**	**278**

How much, if at all, do you think climate change has harmed people in your city or county over the past decade?

Response Options	Percent Response	Response Number (N)
A great deal	20%	56
A moderate amount	46%	129
Only a little	20%	56
Not at all	2%	5
Don't know	12%	32
**Total**	**100%**	**278**

Section B

How much, if at all, do you think climate change is relevant to direct patient care?

Response Options	Percent Response	Response Number (N)
A great deal	24%	67
A moderate amount	42%	116
Only a little	22%	61
Not at all	4%	11
Don't know	8%	21
**Total**	**100%**	**276**

How much, if at all, do you think climate change is affecting the health of your patients?

Response Options	Percent Response	Response Number (N)
A great deal	18%	49
A moderate amount	43%	118
Only a little	18%	50
Not at all	3%	8
Don't know	10%	28
I don't currently see patients	8%	23
**Total**	**100%**	**276**

Those who indicated that they did not currently see patients were skipped ahead to Question C3: "My primary place of work does an effective job minimizing its use of fossil-fuels (e.g., conserving energy/water, recycling equipment, *etc.*)".

In which of the following ways, if any, do you think your patients are currently being affected by climate change, or might be affected in the next 10–20 years? The percentage responses for each question are based on the number of individuals who answered each question.

**Response Options**	**Percent Response**			**Response Number (N)**
**People Are Currently Being Affected**	**Yes**	**Don't Know**	**No**	**Total**
Heat-related effects (e.g., heatstroke, heat exhaustion, cardio-respiratory illness)	75%	14%	11%	236
Vectorborne infection (e.g., Lyme, West Nile, Dengue Fever, Malaria)	58%	26%	16%	232
Diarrhea from food/waterborne illnesses (e.g., Salmonella, Giardia, Cryptosporidia) following downpours or floods	56%	22%	22%	238
Injuries due to severe storms, floods, droughts, fires	88%	8%	5%	240
Air pollution related increases in severity of illness (e.g., asthma, COPD, pneumonia, cardiovascular disease)	88%	10%	3%	242
Increased care for allergic sensitization and symptoms of exposure to plants or mold (visits to office/ER for asthma/allergic symptoms)	80%	13%	8%	240
Mental health problems due to the above	40%	41%	19%	235
**People Will Be Affected in the Next 10–20 Years**	**Yes**	**Don't Know**	**No**	**Total**
Heat-related effects (e.g., heatstroke, heat exhaustion, cardio-respiratory illness)	88%	9%	3%	221
Vectorborne infection (e.g., Lyme, West Nile, Dengue Fever, Malaria)	70%	21%	9%	222
Diarrhea from food/waterborne illnesses (e.g., Salmonella, Giardia, Cryptosporidia) following downpours or floods	67%	23%	10%	222
Injuries due to severe storms, floods, droughts, fires	90%	9%	1%	219
Air pollution related increases in severity of illness (e.g., asthma, COPD, pneumonia, cardiovascular disease)	91%	8%	2%	221
Increased care for allergic sensitization and symptoms of exposure to plants/mold (office/ER visits for asthma/allergy)	86%	11%	4%	222
Mental health problems due to the above	56%	35%	9%	225

Section C

Which of the following, if any, are barriers that prevent you from addressing climate change-related health issues with patients?

Response Options	Percent Response					Response Number (N)
	Strongly Agree	Agree	Neutral	Disagree	Strongly Disagree	Total
Climate change is not occurring	1%	4%	9%	29%	57%	164
My patients would not be interested or knowledgeable enough about climate impacts to discuss this issue	6%	21%	33%	33%	7%	238
Lack of time	27%	42%	18%	9%	4%	239
Lack of knowledge regarding how to approach the issue with my patients	26%	45%	14%	10%	5%	240
Addressing these issues with my patients will not make much difference in their overall health	5%	17%	27%	40%	11%	240
This is not a billable activity	16%	23%	30%	15%	16%	164
Other barriers (please specify)	15%	15%	58%	4%	9%	55

The primary hospital that I admit to is well prepared for climate-related events (e.g., disasters/ emergencies, extreme weather events, increase in certain diseases, *etc.*).

Response Options	Percent Response	Response Number (N)
Strongly agree	15%	34
Agree	35%	82
Neutral	30%	71
Disagree	15%	36
Strongly disagree	5%	12
**Total**	**100%**	**235**

My primary place of work does an effective job minimizing its use of fossil-fuels (e.g., conserving energy/water, recycling equipment, *etc.*).

Response Options	Percent Response	Response Number (N)
Strongly agree	6%	16
Agree	23%	58
Neutral	30%	77
Disagree	34%	88
Strongly disagree	7%	17
**Total**	**100%**	**256**

Teaching about climate change and its association with health impacts should be integrated into medical education.

Response Options	Percent Response	Response Number (N)
Strongly agree	30%	78
Agree	50%	129
Neutral	12%	31
Disagree	5%	14
Strongly disagree	3%	8
**Total**	**100%**	**260**

My medical societies should have a significant advocacy role in relation to climate change and health.

Response Options	Percent Response	Response Number (N)
Strongly agree	28%	71
Agree	48%	125
Neutral	19%	48
Disagree	5%	12
Strongly disagree	1%	2
**Total**	**100%**	**258**

I feel that actions I take in my personal and/or professional life can contribute to effective action on climate change.

Response Options	Percent Response	Response Number (N)
Strongly agree	25%	65
Agree	53%	137
Neutral	17%	43
Disagree	6%	15
Strongly disagree	0%	0
**Total**	**100%**	**260**

Physicians have a responsibility to bring the health effects of climate change to the attention of their patients.

Response Options	Percent Response	Response Number (N)
Strongly agree	24%	63
Agree	51%	132
Neutral	20%	53
Disagree	5%	13
Strongly disagree	0%	0
**Total**	**100%**	**261**

Physicians have a responsibility to bring the health effects of climate change to the attention of the public. This question was only asked on the member survey and not on the leadership survey.

Response Options	Percent Response	Response Number (N)
Strongly agree	25%	43
Agree	46%	78
Neutral	23%	39
Disagree	5%	9
Strongly disagree	0.5%	1
**Total**	**100%**	**170**

Physicians should have a leadership role in encouraging offices, clinics, hospitals to be as environmentally sustainable as possible.

Response Options	Percent Response	Response Number (N)
Strongly agree	34%	88
Agree	47%	121
Neutral	16%	42
Disagree	3%	8
Strongly disagree	0.5%	1
**Total**	**100%**	**260**

Which of the following resources, if any, would be helpful to you?
Policy statements provided by my professional associations.

Response Options	Percent Response	Response Number (N)
Strongly agree	34%	89
Agree	48%	127
Neutral	14%	37
Disagree	3%	8
Strongly disagree	2%	5
**Total**	**100%**	**266**

Which of the following resources, if any, would be helpful to you?
Continuing medical education (CME) on climate change and health.

Response Options	Percent Response	Response Number (N)
Strongly agree	41%	110
Agree	48%	127
Neutral	8%	21
Disagree	3%	7
Strongly disagree	0.5%	1
**Total**	**100%**	**266**

Which of the following resources, if any, would be helpful to you?

Patient education materials.

Response Options	Percent Response	Response Number (N)
Strongly agree	41%	109
Agree	45%	119
Neutral	11%	30
Disagree	3%	7
Strongly disagree	0.5%	1
**Total**	**100%**	**266**

Which, if any, of the following groups will disproportionately experience any negative health effects from climate change? (check all that apply).

Response Options	Percent Response		Response Number (N)
	Yes	No	Total
Young children ages 0 to 4	83%	17%	271
Older children ages 5 to 17	42%	58%	271
Young adults ages 18 to 39	27%	73%	271
Middle aged adults ages 40 to 60	25%	75%	271
Older adults ages 60+	80%	20%	271
People with chronic diseases	88%	12%	271
People living near or below the poverty line	86%	14%	271
People of color	73%	27%	271

Section D

Outside your role as a health professional, to what degree have you personally experienced climate change?

Response Options	Percent Response	Response Number (N)
A great deal	10%	27
A moderate amount	38%	100
Only a little	40%	107
Not at all	6%	15
Don't know	7%	18
**Total**	**100%**	**267**

How big of an effort should the U.S. make to reduce the potential impacts of climate change (prevention)?

Response Options	Percent Response	Response Number (N)
A large-scale effort, even if it has large economic costs	55%	146
A medium-scale effort, even if it has moderate economic costs	38%	100
A small-scale effort, even if it has small economic costs	7%	18
No effort	1%	3
**Total**	**100%**	**267**

How big of an effort should the U.S. make to protect people from the potential harmful health effects caused by climate change (preparedness)?

Response Options	Percent Response	Response Number (N)
A large-scale effort, even if it has large economic costs	56%	149
A medium-scale effort, even if it has moderate economic costs	37%	98
A small-scale effort, even if it has small economic costs	7%	19
No effort	1%	2
**Total**	**100%**	**268**

Section E

What is your primary work setting?

Response Options	Percent Response	Response Number (N)
Outpatient (clinical)	49%	126
Hospital (clinical)	29%	74
Non-clinical Administrative	8%	20
Other clinical	8%	21
Other non-clinical	6%	16
Retired	0.5%	1
**Total**	**100%**	**258**

Which is, or if retired was, your primary work location?

Response Options	Percent Response	Response Number (N)
Urban	69%	167
Suburban	22%	52
Rural	5%	12
Multiple	4%	10
**Total**	**100%**	**241**

In which U.S. State do you (or did you) work?

Response Options	Percent Response	Response Number (N)
Alabama	1%	3
Arizona	1%	2
California	8%	20
Colorado	1%	3
Connecticut	0.5%	1
Delaware	1%	2
District of Columbia	6%	14
Florida	4%	10
Georgia	10%	26
Hawaii	0.5%	1
Illinois	3%	7
Indiana	2%	5
Kansas	0.5%	1
Kentucky	0.5%	1
Louisiana	4%	9

Maryland	10%	24
Massachusetts	4%	9
Michigan	3%	8
Minnesota	1%	2
Mississippi	1%	2
Missouri	3%	8
Nevada	1%	3
New Jersey	2%	5
New Mexico	0.5%	1
New York	7%	18
North Carolina	4%	9
Ohio	4%	9
Pennsylvania	2%	6
South Carolina	0.5%	1
Tennessee	3%	7
Texas	7%	17
Virginia	4%	9
Wisconsin	2%	6
**Total**	**100%**	**249**

Which of the following degrees or certifications do you hold? (check all that apply).

Response Options	Percent Response	Response Number (N)
M.D.	81%	215
Ph. D.	2%	4
PA/CRNP	0.5%	1
Master's Degree	1%	2
Other clinical degree	1%	3
Other non-clinical degree	2%	4
RN	0.5%	1
Multiple degrees	13%	35
**Total**	**100%**	**265**

33 out of 35 respondents who hold multiple degrees indicated that one of the degrees was an M.D.

Which best describes your medical training?

Response Options	Percent Response	Response Number (N)
Internal Medicine/Family Medicine	29%	74
Pediatrics	14%	36
OB/Gyn	11%	28
Surgery	5%	12
Other Specialty	28%	71
Other Practice	4%	9
Multiple	10%	26
**Total**	**100%**	**256**

If you are in clinical practice, what proportion of your patient population is non-white?

Response Options	Percent Response	Response Number (N)
Less than 25%	21%	49
26%–50%	26%	60
51%–75%	21%	49
More than 75%	31%	71
**Total**	**100%**	**229**

If you are in clinical practice, what proportion of your patient population is covered by the following insurance arrangements?

Response Options	Percent Response		Response Number (N)
	**Less than 50%**	**More than 50%**	**TOTAL**
Self-Pay	99%	1%	126
Medicare	83%	17%	134
Medicaid	76%	24%	137
Private	72%	29%	137
Other	68%	32%	25

What is your gender?

Response Options	Percent Response	Response Number (N)
Female	62%	163
Male	40%	97
Female and Male	0.5%	1
Prefer not to answer	1%	2
**Total**	**100%**	**263**

What is your age?

Response Options	Percent Response	Response Number (N)
18–30	5%	14
31–50	38%	100
51–65	43%	111
66 or older	14%	36
**Total**	**100%**	**261**

Please specify your ethnicity:

Response Options	Percent Response	Response Number (N)
Hispanic or Latino/a	1%	3
Not Hispanic or Latino/a	99%	252
**Total**	**100%**	**255**

Please specify your race (check all that apply):

Response Options	Percent Response	Response Number (N)
American Indian or Alaskan Native	1%	3
Asian	0%	0
Black or African American	95%	250
Native Hawaiian or other Pacific Islander	0%	0
White	0%	0
Multiple/Bi-racial	3%	9
**Total**	**100%**	**262**

Would you like to assist in education or advocacy focused on climate and health in your community? (This question requested that the individual leave his/her name on a separate page.)

Response Options	Percent Response	Response Number (N)
Yes	33%	85
No	67%	172
**Total**	**100%**	**257**

Survey Group (not a response option).

Response Options	Percent Response	Response Number (N)
Leadership Survey March 2014	36%	101
Member Survey May 2014	64%	183
**Total**	**100%**	**284**

© 2014 by the authors; licensee MDPI, Basel, Switzerland. This article is an open access article distributed under the terms and conditions of the Creative Commons Attribution license (http://creativecommons.org/licenses/by/4.0/).
